# Supplementary material for: Self-Powered and Flexible Triboelectric Sensors with Oblique Morphology towards Smart Swallowing Rehabilitation Monitoring System
Source: Materials (Basel). 2022 Mar 18;15(6):2240. doi: 10.3390/ma15062240 (PMC8954625; doi:10.3390/ma15062240)
Supplement: Supplementary file 1 [file materials-15-02240-s001.zip › materials-1562675-supplementary.pdf]

Supporting Information

Self-powered and flexible triboelectric sensors with the oblique morphology towards smart swallowing rehabilitation monitoring system

Jonghyeon Yun[1,3], Hyunwoo Cho[1,3], Jihyeon Park[1,3] and Daewon Kim[2,3]*

[1] Department of Electronics and Information Convergence Engineering, Kyung Hee University, 1732 Deogyeong-daero, Giheung-gu, Yongin 17104, Republic of Korea

[2] Department of Electronic Engineering, Kyung Hee University, 1732 Deogyeong-daero, Giheung-gu, Yongin 17104, Republic of Korea

[3] Institute for Wearable Convergence Electronics, Kyung Hee University, 1732 Deogyeong-daero, Giheung-gu, Yongin 17104, Republic of Korea

* Corresponding authors.

Email address: daewon@khu.ac.kr (D. Kim)

Table of contents

1. **The electrical output generated from the TSR after conducting the tilted reactive ion etching according to the tilted angle range in 55° to 70°.**
2. **The electrical output generated from the TSR as the pressure increases.**
3. **The morphology of the PTFE film according to the RIE**
4. **The hydrophobicity of the PTFE film**
5. **The electrical output voltage and current.**
6. **The surface of the PTFE film after conducting the tilted RIE observed by the SEM with the tilted sample stage of 0°, 15°, and 30°.**
7. **The surface of the PTFE film after conducting the tilted RIE observed by the SEM with the tilted sample stage of 45°, 60°, and 75°.**
8. **The formed oblique morphology after conducting the tilted RIE.**

1. The electrical output generated from the TSR after conducting the tilted reactive ion etching according to the tilted angle range in 55° to 70°.

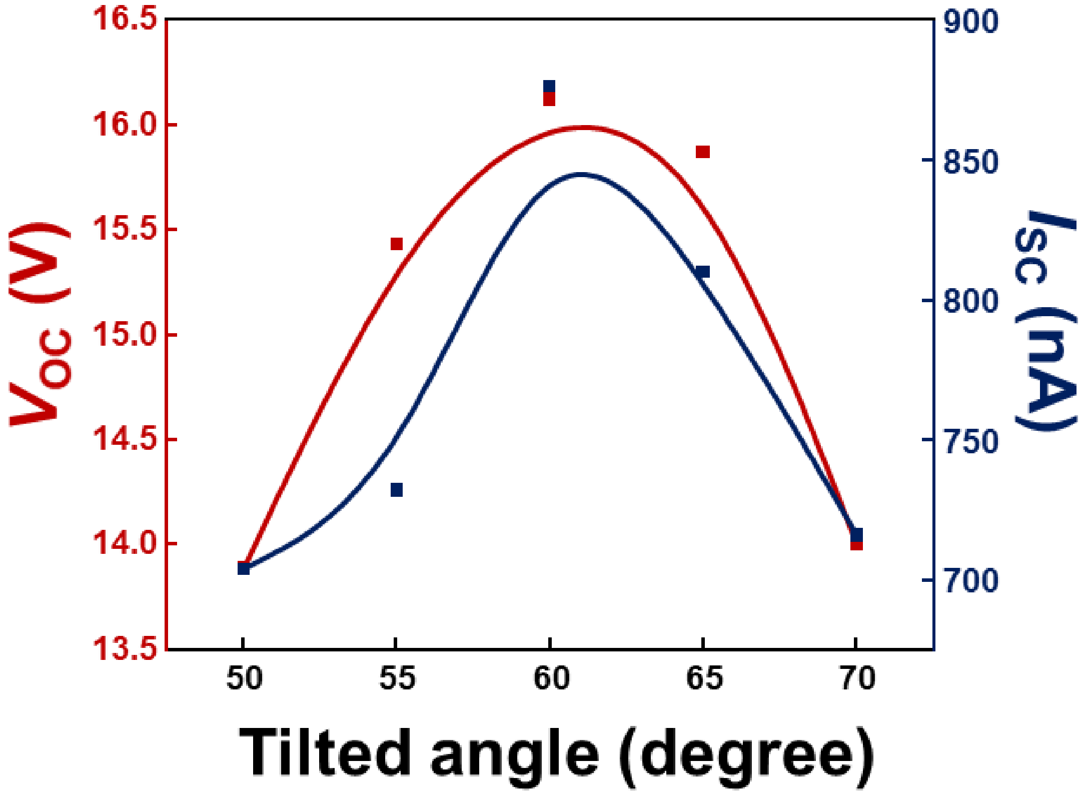

Figure S1. The generated electrical output after conducting tilted RIE with the angle of 50°, 55°, 60°, 65°, and 70°.

2. The electrical output generated from the TSR as the pressure increases.

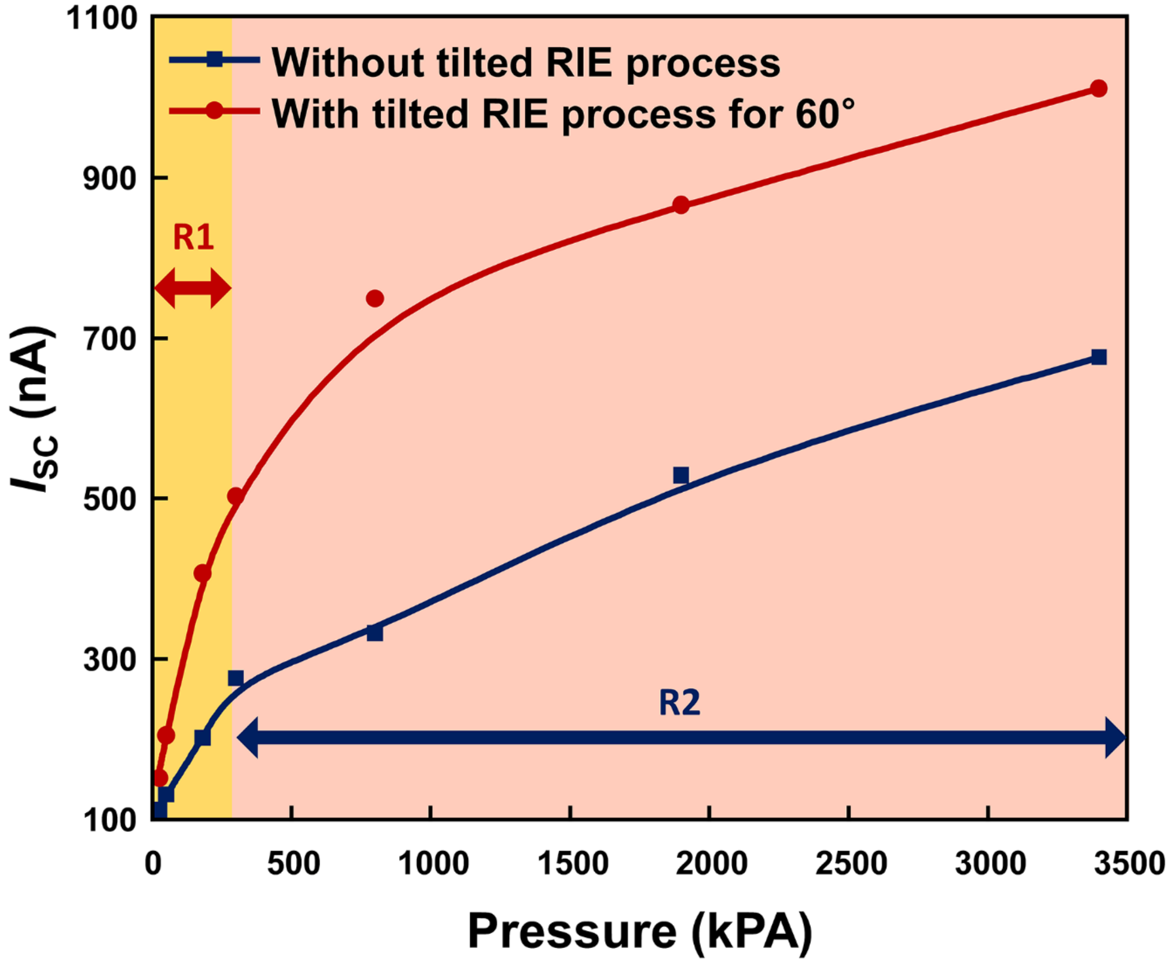

Figure S2. The generated short-circuit current according to the pressure.

3. The morphology of the PTFE film according to the RIE

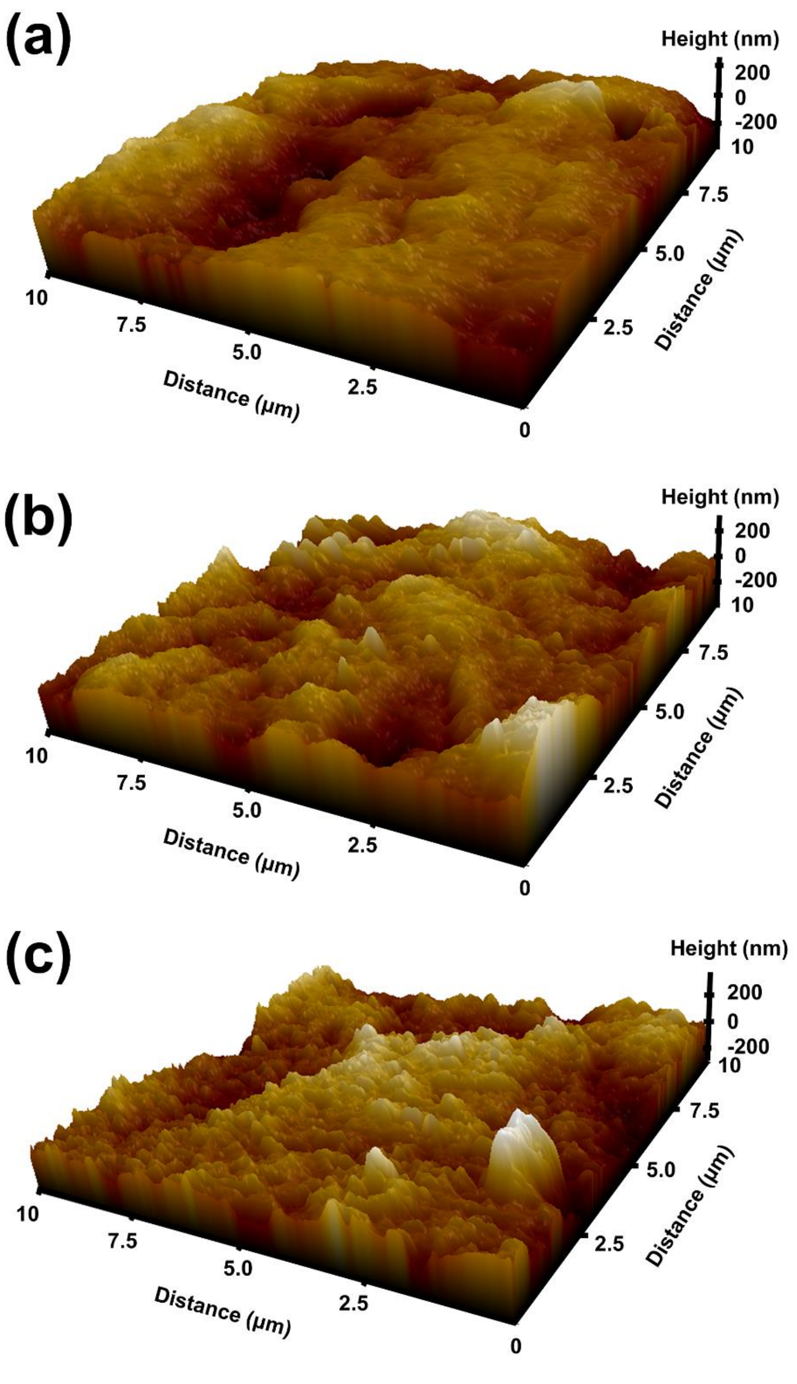

Figure S3. The morphology formed after conducting tilted RIE process to the PTFE film. (a) bare PTFE film. (b) PTFE film after tilted RIE with angle of 30°. (c) PTFE film after tilted RI E with angle of 60°.

4. The hydrophobicity of the PTFE film

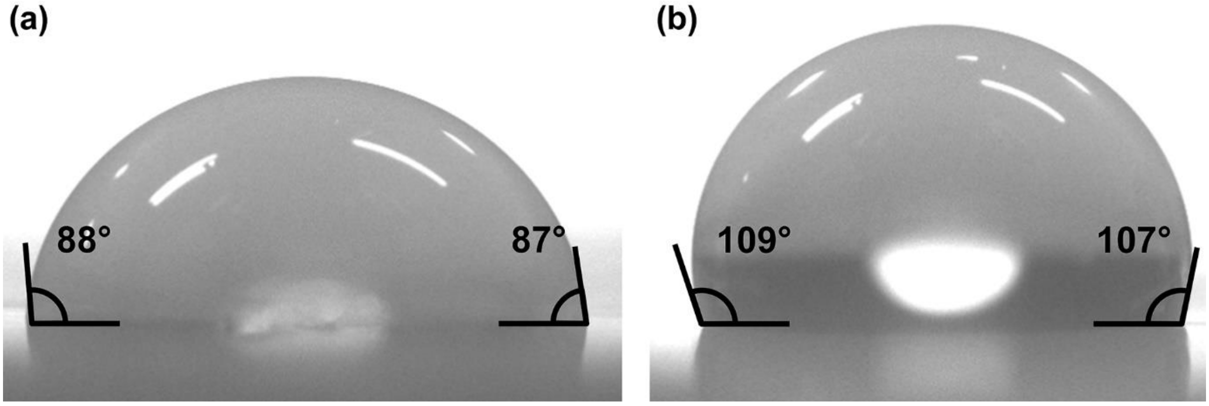

Figure S4. (a) Contact angle of bare PTFE film and (b) RIE-treated PTFE film with tilted angle of 60°.

5. The electrical output voltage and current.

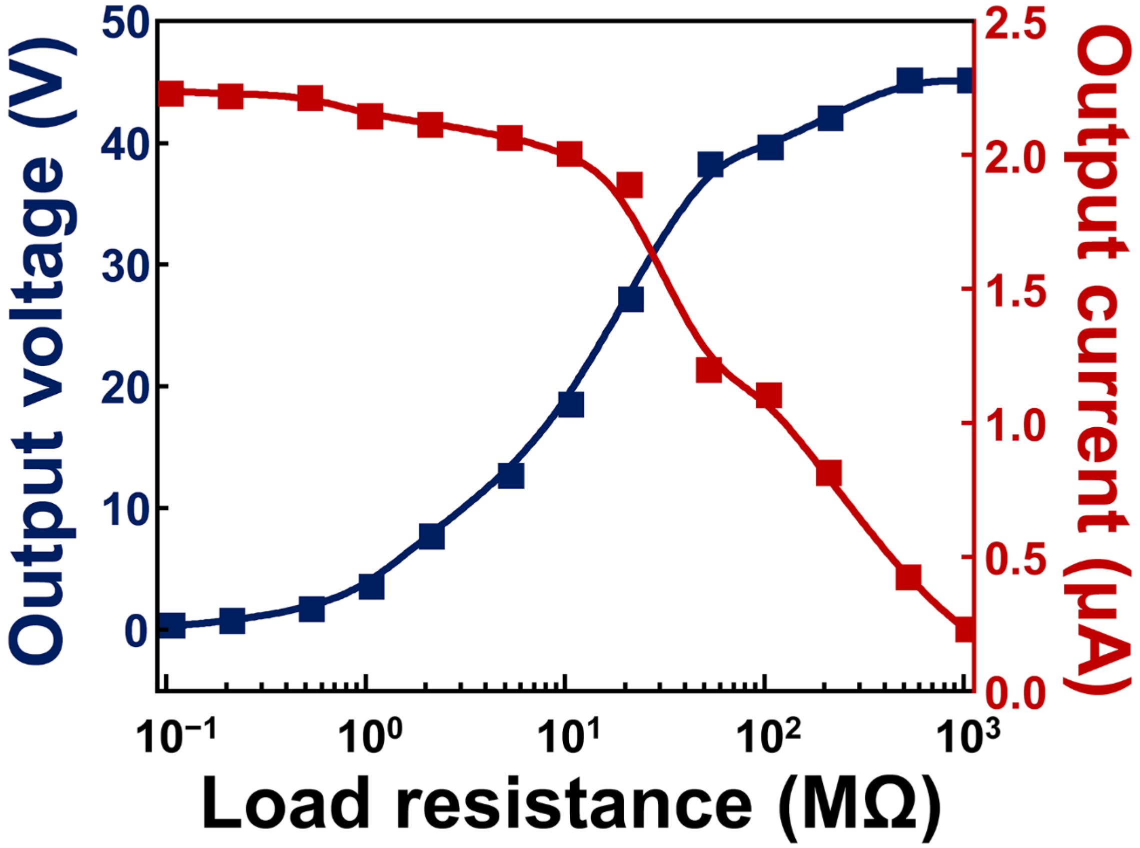

Figure S5. The output voltage and current with the various load resistances.

6. The surface of the PTFE film after conducting the tilted RIE observed by the SEM with the tilted sample stage of 0°, 15°, and 30°.

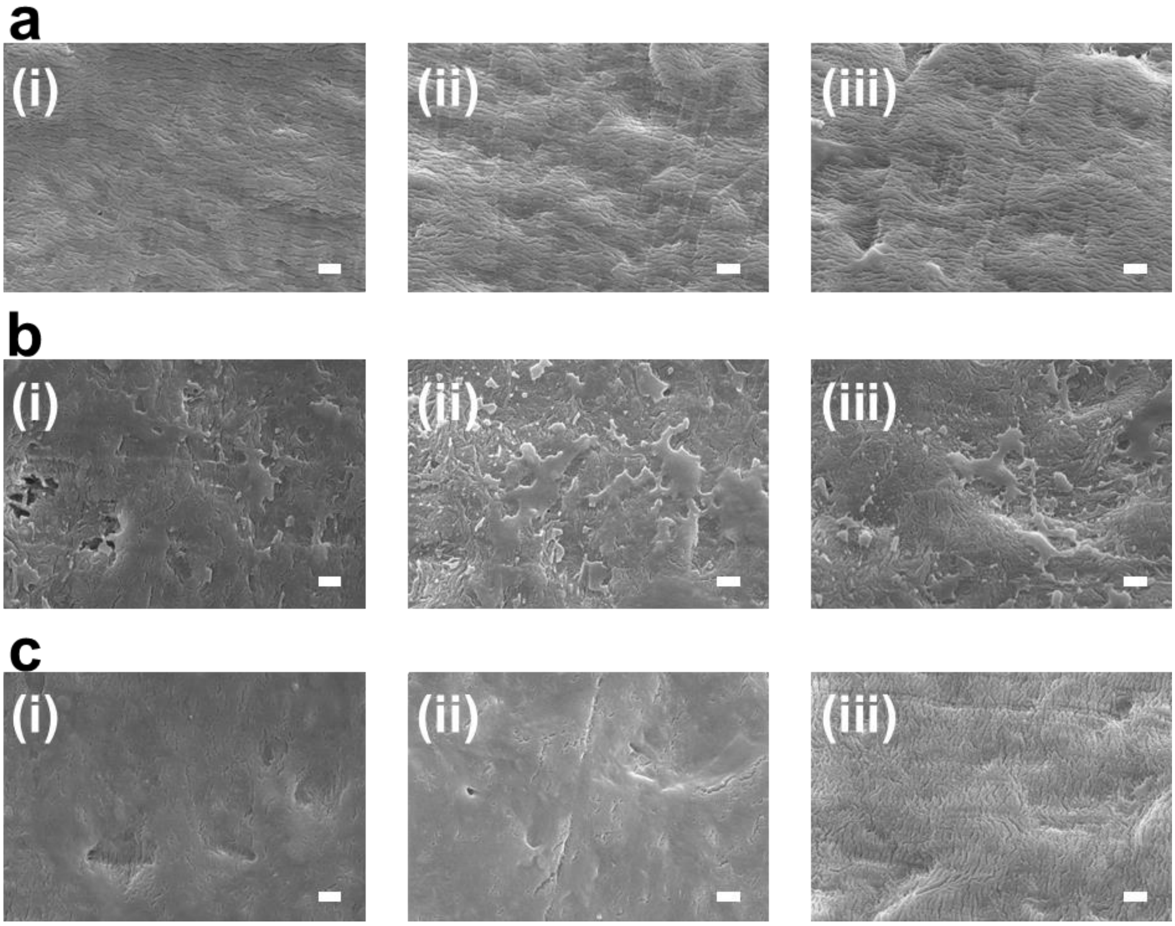

Figure S6. The surface of the PTFE film with the tilted reactive ion etching with the tilted angle of (a) 0°, (b) 15°, and (c) 30° observed by the SEM with the tilted angle of the sample stage of (i) 0°, (ii) 15°, and (iii) 30°, respectively. The scale bars indicate 500 nm.

7. The surface of the PTFE film after conducting the tilted RIE observed by the SEM with the tilted sample stage of 45°, 60°, and 75°.

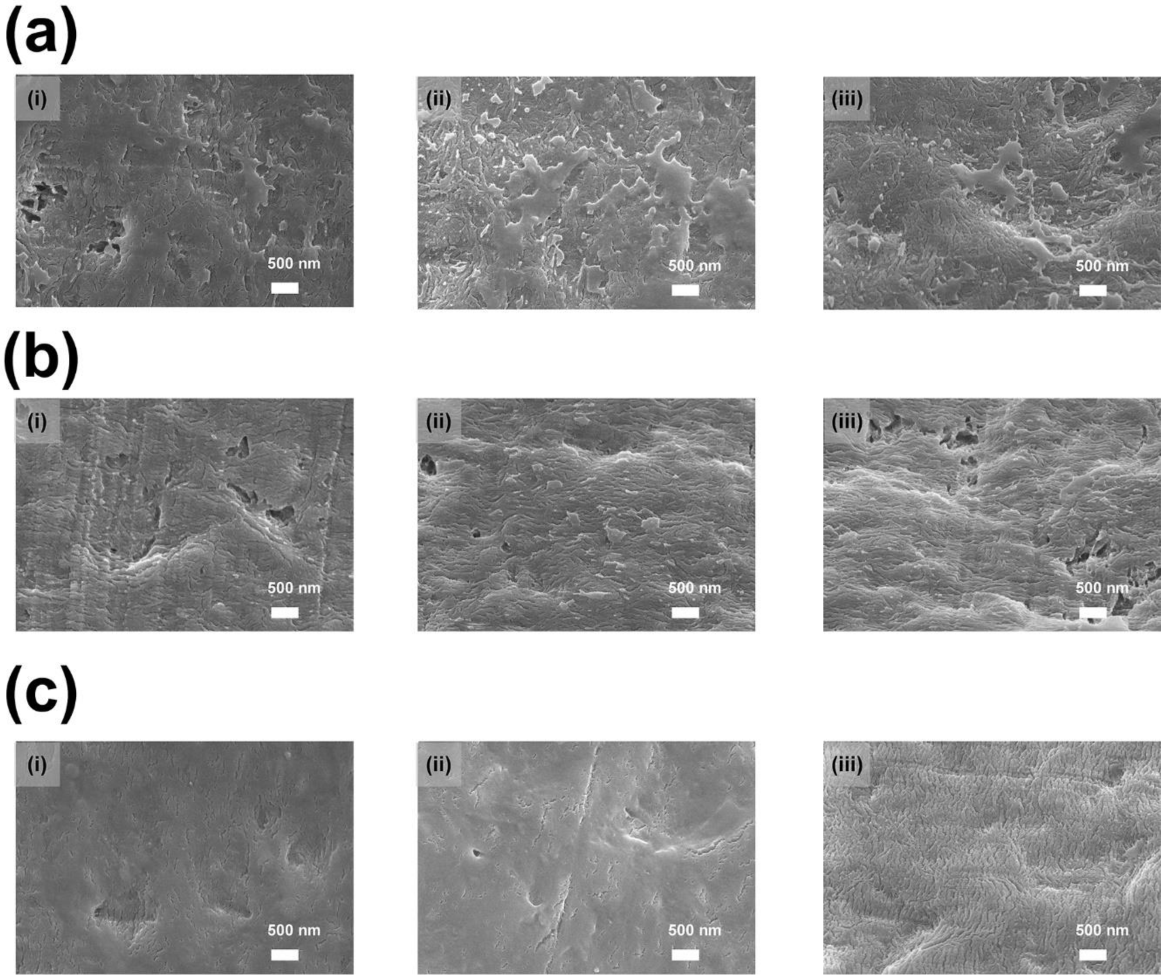

Figure S7. The surface of the PTFE film with the tilted reactive ion etching with the tilted angle of (a) 45°, (b) 60°, and (c) 75° observed by the SEM with the tilted angle of the sample stage of (i) 0°, (ii) 15°, and (iii) 30°, respectively. The scale bars indicate 500 nm.

8. The formed tilted nanostructures after conducting the tilted RIE

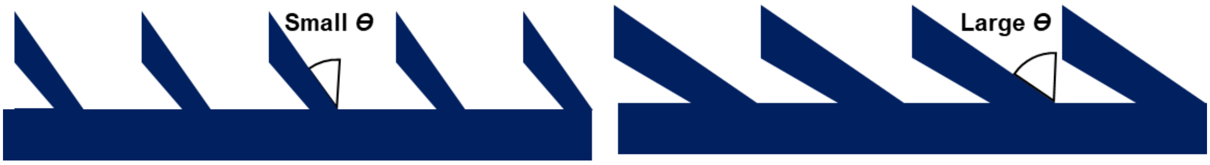

Figure S8. The oblique morphology formed after conducting tilted RIE process to the PTFE f

ilm.
